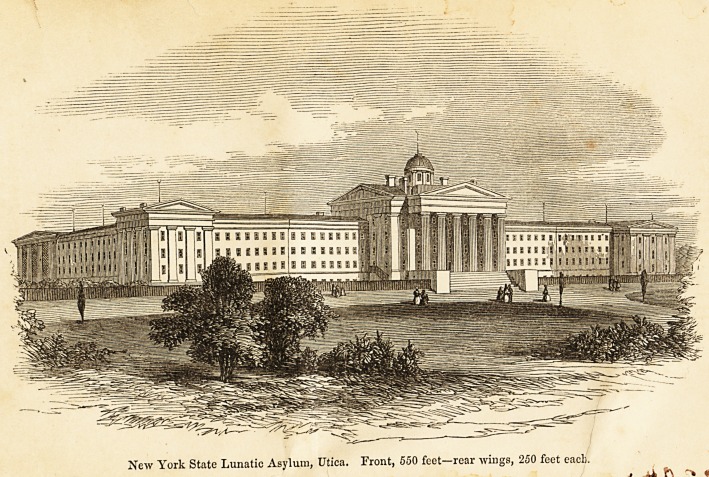# Insanity in the State of New York

**Published:** 1848-01-01

**Authors:** 


New York State Lunatic Asylum, Utica. Front, 550 feet—rear wings, 250 feet each.

2204.

Art. VIII.—*Fourth Annual Report of the Managers of the State Lunatic Asylum, near Utica, New York, U. S. Feb.* 2, 1847.

This valuable and interesting Report is drawn up chiefly by Dr. Brigham, the superintendent and head physician of the splendid establishment erected within the last few years for the insane of the state of New York, and at the expense of the legislature, near the flourishing central city of Utica. The building is constructed to accommodate six hundred patients; and both its plan and system of government were arranged after a careful examination of no less than fourteen out of the twenty similar institutions in the United States. We may therefore expect the Annual Reports of this asylum, which are made to the Government in accordance with an act of the State, to exhibit a fair picture of the results afforded by the most approved management and treatment of the insane which our kinsmen on the other side of the Atlantic have been able to attain.

The present Report is for the year ending November 30, 1846; during which period there were 622 patients in the asylum, composed of 306 males and 316 females, which numbers comprise 143 males and 142 females remaining since the preceding year, the current admissions being 163 males and 174 females. The patients discharged during the year were,—

| | Males. | Females. |
|---|---|---|
| Recovered . . . . | 65 | 68 |
| Improved . . . . | 26 | 34 |
| Unimproved . . . . | 15 | 18 |
| Deaths . . . . . | 13 | 9 |
| Total . . | 119 | 129 |

Leaving for the ensuing Report 374 patients, composed of equal numbers of males and females.

It will be seen that the per centage (21·38) of recoveries in the above table is among the lowest that ever occur, being scarcely more than one in five; and this although the institution has been remarkably exempt from sickness, no fever, nor any contagious or epidemic complaint having ever prevailed in it. The general average, however, for the four years during which it has existed, presents a much more favourable aspect, being 38·35 per cent., or considerably more than one in three, which M. Esquirol calculates to be about the absolute number of recoveries in cases of insanity. The following table presents the statistics of the asylum from its opening in January 1843, to the close of the last Report:—

| | | |
|---|---|---|
| Total number admitted . . | | 1181 |
| Discharged, recovered . . . . | 453 | |
| „ improved . . . . | 199 | |
| „ unimproved . . | 89 | |
| Died . . . . . . | 66 | |
| | | 807 |
| Remaining . | | 374 |

Insanity is not a very fatal disease; and the tables show a conformity in this respect of the American with our European experience. The per centage of deaths over the four years is only 5·58, while that for last year is considerably less, being only 3·53. The recoveries and improvements, however, taken together, are very much greater for the whole period, being far above the one-half, than for the last year, which do not amount to one-fourth. But this is only in accordance with the well known capricious nature of the curability of insanity; one year exhibiting most favourable results, while the following may show a lamentable falling off, without there being assignable any sufficient explanation, derived from changes in the atmospherical phenomena, or connected with any kind of popular excitement.

It cannot but be a matter of the highest gratification to every humane mind to perceive, as the general result of the statistical tables of insanity hitherto published, that at least one half of the miserable subjects of this most fearful calamity are capable, under proper treatment, of recovery or improvement. This cannot be said of many of the commonest diseases which afflict mankind. And yet how sedulously will friends and relatives watch the earliest germinations of consumption, for instance, and persevere in every remedy till the last gasp; while the caprices of temper or the changes in character, which are the shadows of coming events far more frightful and deplorable than the excavations of scrofula or the agonies of cancer, are from ignorance unheeded, or concealed from shame; and when the necessary seclusion has at length been sought for, how impatient for results, how anxious for interference, how wearied of expense, or desirous of change! On this subject we recommend to the attention of the public the following observations from the Report:—

"A complaint made at most of the institutions for the cure of the insane, that patients are removed too soon, we have very rarely had occasion to make. This we suppose is mainly to be attributed to the law of this state respecting this asylum, that 'no patient shall be admitted for a shorter period than six months.' That is, patients shall not be removed in less than six months contrary to the advice of the officers of the asylum, who not having any interest whatever in patients remaining at the institution longer than for their good, will advise to their timely removal. For the good of the insane, we believe such a regulation should be adopted in other institutions, and that patients should not be received on trial merely for three months, as is often the case. According to our experience, recent and curable cases do not generally recover in three months, but a majority do in four or five months. Consequently, if patients are removed at the end of one quarter, when beginning to convalesce, they are very liable to relapse, while if they could be permitted to remain but a month or two longer, permanent recovery might be secured.

"The law of this state is a wise regulation in other respects. Not unfrequently the guardians of insane persons are forced by public opinion, and contrary to their own wishes, to place those under their charge at an asylum for the insane. But they are sometimes not disposed to give them a fair trial of curative treatment, and frequently will not, unless there is such a regulation as we have referred to; consequently patients thus removed in a short time are not materially improved; the benefits and utility of institutions for the cure of the insane are decried, and the worst effect is, to deter other patients that might be restored, from being sent to them. But, as we have said, we have no complaint to make in this respect, as our patients are generally permitted to remain until they have had a good trial of curative means.

"We always, however, discharge patients as soon as we think it for their good, and safe to do so. But the exact time when it is best for patients, who appear to have recovered, to leave an asylum and return to their homes, is often exceedingly difficult to determine. Sometimes, no doubt, a patient is kept a little longer than is absolutely necessary, but most frequently mistakes are the other way, and patients are permitted to leave too soon; not, perhaps, before they are rational, but before the condition of the brain and nervous system has become such as to enable them to engage in business, and take an active part in society without injury. Such very frequently relapse, and having failed to recover permanently in a few weeks at a lunatic asylum, they are ever after kept at home, usually confined, and soon become incurable maniacs.

"We deem it of great importance that the public should have correct views on this subject, and fully understand the necessity of enabling every person affected by insanity to have a *full trial of curative means in the early stage of the disease*, as generally such cases, if properly treated, are restored. Whenever the community realize this, there will be a great diminution of the numbers of the incurable insane of the country."—Pp. 18, 19.

It is an interesting question, in a prophylactic point of view, to determine, if possible, at what season of the year maniacal patients are most liable to the attack. The following table is given in the Report, though it is presumed to contain many errors, of the season of the year when the patients were supposed to have become insane:—

| Jan. | Feb. | March. | April. | May. | June. | July. | Aug. | Sept. | Oct. | Nov. | Dec. | Unknown. | Total |
|---|---|---|---|---|---|---|---|---|---|---|---|---|---|
| 82 | 70 | 109 | 86 | 113 | 100 | 86 | 82 | 97 | 113 | 83 | 76 | 84 | 1181. |

But the only inference that can be drawn from this is, that attacks are a little more numerous in the variable weather of spring and autumn, than in mid-summer and in winter.

TABLE OF AGES WHEN INSANITY COMMENCED.

| Under 20 years | From 20 to 25 | From 25 to 30 | From 30 to 35 | From 35 to 40 | From 40 to 45 | From 45 to 50 | From 50 to 55 | From 55 to 60 | From 60 to 65 | From 65 to 70 | From 70 to 75 | Over 80 |
|---|---|---|---|---|---|---|---|---|---|---|---|---|
| 123 | 241 | 198 | 151 | 148 | 108 | 78 | 46 | 40 | 30 | 14 | 3 | 1 |

From this it appears that ten per cent. were attacked up to the age of twenty; for the next ten years the ratio is nearly quadrupled; it then diminishes during the next ten years to about twenty-five per cent.; in the next five years, or in the prime of life, it returns to about the ratio of youth, continuing gradually to decrease till after the grand climacteric, when it is reduced to a fraction more than one per cent.

From a table illustrative of the occupations and civil condition of the insane, it appears that the farmers and labourers, whose natural and healthy employments might be thought to bestow almost an exemption from this malady, afford no less than twenty per cent. of the whole number. The mercantile class, whose anxieties and feverish speculations might be supposed to irritate the nervous system far more than the unvaried and steady occupations of the farmer, yield only three per cent.; while among the female patients, the monotonous tone of an indolent or merely housewifely occupation seems to afford a parallel to the agricultural, their numbers bearing as high a proportion to the whole as forty-two per cent. Does it not seem to follow, that the energetic employment of all the powers of the brain, which is the necessary condition of a life of trade and business, is more favourable to the continuance of mental health than the partial employment of some faculties, and the stagnant condition of many others, as in the farming and domestic classes? The medical profession supplied ten patients, and the clerical 6; but what will be considered surprising is, that the civil condition of married and single give respectively almost equal proportions, the former being 545 and the latter 564.

There is also a table given of supposed causes; but the information was supplied merely by the friends of the patients, who are often unwilling or mistaken in assigning the true ones. The most numerous are stated to be "unknown, ill health, or of doubtful origin." To "intemperance" are assigned only thirty-five cases; but when we recollect the habits of dram drinking, so commonly attributed to the American people, under such a variety of ridiculous names, we cannot help thinking it sufficiently proven that this table of causes is of no value whatever. Religious anxiety is given as a cause in sixty-two male and sixty female cases, being ten per cent. of the whole; and we can only wonder that it is not double or triple, in a country so notorious as the United States for such an exhibition as is assigned in one case as the supposed cause of the attack—viz. "preaching sixteen days and nights." Political excitement, disappointed ambition, and extravagant opinions, furnish only sixteen cases.

Dr. Brigham gives a table "On the frequency of the pulse of the insane," which we shall quote entire:—

"Our observations commenced at the Retreat for the insane at Hartford, Conn., in 1840, and continued at this asylum, on the frequency of the pulse of the insane, have furnished us the following results:—

*Pulse of the Insane.*

| | | | | | |
|---|---|---|---|---|---|
| From | 40 | to | 50 | in | 8 |
| " | 50 | " | 60 | " | 22 |
| " | 60 | " | 70 | " | 183 |
| " | 70 | " | 80 | " | 233 |
| " | 80 | " | 90 | " | 466 |
| " | 90 | " | 100 | " | 144 |
| " | 100 | " | 110 | " | 124 |
| " | 110 | " | 120 | " | 54 |
| | | | | | 1234 |

Of the physical formations that may be imagined to predispose to insanity, Dr. Brigham has specially noticed the size of the head. He took the measurements very carefully of no less than 1163 patients; but as a general result, there is no material difference in the size of the sane and the insane. The measurements were taken round the circumference, from one meatus auditorius over the head to the other, and from the root of the nose to the occipital protuberance.

| In Men. | Circum. | Ears. | Antero-post. | In Women. | Circum. | Ears. | Antero-post. |
|---|---|---|---|---|---|---|---|
| 8 | 24 | $14\frac{1}{2}$ | $15\frac{1}{2}$ | 50 | $22\frac{1}{2}$ | 14 | $14\frac{1}{2}$ |
| 314 | Average do. 23 | 14 | $15\frac{1}{3}$ | 159 | Average do. 22 | $13\frac{1}{2}$ | $14\frac{1}{2}$ |
| 282 | $21\frac{1}{2}$ | $13\frac{1}{6}$ | 14 | 350 | 21 | 13 | $13\frac{6}{10}$ |

In five sane men the sizes were something less; and in forty others, pretty nearly identical; and in thirty-seven sane women quite so.

The weight and height of patients were also taken on admission, but there seems nothing worthy of notice in these circumstances, except the valuable fact, that "in recent cases of insanity, when patients begin to increase in flesh, we usually predict recovery, especially if at the same time there is some improvement of the mind. On the contrary, where the digestion, and sleep, and appetite are natural, and the patient increases in flesh, without any diminution of insanity, there is little hope of recovery; also, if the appetite continues good, and emaciation increases, there is reason to fear an unfavourable result."

The report affords no information relative to the medical treatment that was adopted, but the moral management seems to have been very judicious and beneficial. There is a very satisfactory account of the results of the out-door labour of the men on the farm attached to the institution. Hay, corn, oats, potatoes, and all kinds of kitchen vegetables, have been produced in abundance; and it is anticipated that the shops—for it is part of the plan to provide the patients with employment at their different trades—will soon supply all the clothing and furniture used at the establishment. Religious services are constantly performed on Sundays throughout the year, with great advantage, as well as monthly concerts; and frequent opportunities are taken of instructing those in the employment of the institution respecting the nature of insanity, and their duties towards the patients. Schools are opened during the winter season, and great confidence is expressed in their utility; some striking cures are even spoken of as the apparent result of the mental exertion they induce. But the amusements are too much restricted, for card-playing, and other games in which there is no bodily exercise, are discouraged; but theatrical exhibitions of original plays, and even weekly debating societies, are encouraged! These, with a good library, abundance of daily and weekly newspapers, a few animals, and a greenhouse, in which upwards of a thousand plants are kept, are the means employed to interest and amuse.

On the subject of hereditary predisposition to insanity, the following observations occur:—

"There is nothing in connexion with the study of insanity more deserving of attention than the tendency of this disease to be transmitted from parents to their offspring. The fact is most unquestionable, and we are of opinion that it has more influence in producing that disease than all other causes combined. It does not of itself excite the disease, but when it strongly exists a trivial cause will develop it. Thus most of the supposed exciting causes in the foregoing table, would of themselves be inoperative, if there was not an inherited constitutional tendency to insanity.

"Sometimes the children of an insane parent are, however, exempt from the disease, while it appears in the grandchildren. Contrary to the opinion of many, we have found this inherited form of insanity as curable as any other, though the subjects of it are very liable to relapse, and from slight and various causes. Sometimes a little sickness, a slight fever, or severe cold, and at others a little mental disturbance, such as the loss of relatives, or property, or religious anxiety, excite it. We have known individuals thus predisposed to insanity, have repeated attacks, and each time from a different exciting cause.

"Of 1181 patients who have been at this asylum—viz., 594 men and 587 women, 315 were known to have insane relatives. That many of the others were thus predisposed we do not doubt, but we were not able to learn anything respecting their relatives. 175 were known to have insane parents—viz., 79 men and 96 women.

"It would appear from our inquiries, and they have been very carefully conducted, that insanity is a little more likely to be transmitted by the mother than by the father, and that mothers are considerably more likely to transmit it to daughters than to sons; while the fathers most frequently transmit it to sons. Thus, out of 79 men, 42 had insane fathers and 35 insane mothers, and in two instances both parents were deranged; while of 96 women, 37 had insane fathers and 56 insane mothers, and three inherited a predisposition to insanity from both parents.

"Sometimes the children of an insane parent do not inherit any tendency to insanity. In such instances, the exemption appears to be in consequence of inheriting the constitution and temperament of the parent not insane. When, however, the children resemble in personal appearance the insane parent, and manifest the same peculiarities of feelings and temper, there is reason to apprehend they will be more or less disposed to the disorders of the parent they resemble.

"These facts cannot fail to arrest the attention, not only of those who have relatives and friends that are insane, but of every philanthropist; and be taken into consideration in forming matrimonial alliances, and be duly regarded in the physical and moral education of those thus liable by inheritance to insanity.

"The early education of all such requires much attention. Great pains should be taken to form a character not subject to strong emotions, to passion, and caprice. Among the most frequent causes of insanity, in those not predisposed to it, is the over-indulgence of the appetites and passions in early life; and to those who inherit a tendency to this disease, such a course is highly pernicious.

"The utmost attention should be given to securing a good bodily constitution. Such children should be confined but little at school; they should be encouraged to run about the fields, and to take much exercise in the open air, and thus ensure the equal and proper development of all the organs of the body. They should not have the intellect unduly tasked. Very early cultivation of the mind, and the excitement of the feelings by the strife for the praise and the honour awarded to great efforts of mind and memory, are injurious to all children, and to those who inherit a tendency to nervous diseases or insanity, most pernicious."—Pp. 35—37.

## Figures and Tables

**Figure f1:**